# Novel implementation of conditional co-regulation by graph theory to derive co-expressed genes from microarray data

**DOI:** 10.1186/1471-2105-9-S9-S7

**Published:** 2008-08-12

**Authors:** Arun Rawat, Youping Deng

**Affiliations:** 1 University of Southern Mississippi, Hattiesburg, MS-39406, USA

## Abstract

**Background:**

Most existing transcriptional databases like Comprehensive Systems-Biology Database (CSB.DB) and Arabidopsis Microarray Database and Analysis Toolbox (GENEVESTIGATOR) help to seek a shared biological role (similar pathways and biosynthetic cycles) based on correlation. These utilize conventional methods like Pearson correlation and Spearman rank correlation to calculate correlation among genes. However, not all are genes expressed in all the conditions and this leads to their exclusion in these transcriptional databases that consist of experiments performed in varied conditions. This leads to incomplete studies of co-regulation among groups of genes that might be linked to the same or related biosynthetic pathway.

**Results:**

We have implemented an alternate method based on graph theory that takes into consideration the biological assumption – conditional co-regulation is needed to mine a large transcriptional data bank and properties of microarray data. The algorithm calculates relationships among genes by converting discretized signals from the time series microarray data (AtGenExpress) to output strings. A 'score' is generated by using a similarity index against all the other genes by matching stored strings for any gene queried against our database.

Taking carbohydrate metabolism as a test case, we observed that those genes known to be involved in similar functions and pathways generate a high 'score' with the queried gene. We were also able to recognize most of the randomly selected correlated pairs from Pearson correlation in CSB.DB and generate a higher number of relationships that might be biologically important. One advantage of our method over previously described approaches is that it includes all genes regardless of its expression values thereby highlighting important relationships absent in other contemporary databases.

**Conclusion:**

Based on promising results, we understand that incorporating conditional co-regulation to study large expression data helps us identify novel relationships among genes. The other advantage of our approach is that mining expression data from various experiments, the genes that do not express in all the conditions or have low expression values are not excluded, thereby giving a better overall picture. This results in addressing known limitations of clustering methods in which genes that are expressed in only a subset of conditions are omitted.

Based on further scope to extract information, ASIDB implementing above described approach has been initiated as a model database. ASIDB is available at .

## Background

It has been established that co-regulated genes exhibit similar expression patterns as a norm and not as an exception [1]. Microarray allows sensitive, detection of small differences in transcript abundance [2], therefore it is utilized extensively to study co-regulation of genes. The gene expression imprinted in the microarray is the manifestation of the pathway activity undergone by the organism, and every gene performs its obligatory function in various pathways [3]. The genes do not co-express with the same set of genes all the time, but under various conditions will be expressed with different sets of genes termed as conditional coregulation [3,4]. Each gene is estimated to interact with four to eight other genes and associated with 10 biological functions [5]. The DNA microarray therefore assists in measuring the difference in transcriptional activity by comparing their mRNA levels under different experimental conditions like developmental stages, stress, or osmotic shock [6]. Various approaches exist for interpretation of relative gene expression. One of the basic strategies is to set the expression level to three states, i.e. underexpression, baseline and overexpression using a fold change cutoff like two times fold change against the control [7]. Other strategies include setting thresholds representing significant changes between subsequent timepoints and storing in bins [8], an adaptive procedure that takes gene-specific variation into consideration to derive the gene expression in different states [7]. Further, microarray data consists of variations generally termed as interesting variations, which are biologically important, and are superimposed by "obscuring variation" or systematic variation [9].

We have developed an alternate algorithm based on graph theory that takes a discretized expression matrix as input and emits output string. Not all genes are expressed all the time and are required at different developmental and maturation phases of plant [10]. We therefore categorized each differentially expressed gene as "ON/OFF" from every experiment against its control in three states: overexpression (+1), underexpression (-1) and no expression (0). We utilized the regularized t-test to derive differentially expressed genes to overcome low replicates and extract meaningful biological variances [11]. We derived a discretized expression matrix for all the genes for various time series experimental conditions from differentially expressed genes. The similarity between two (or more) discretized vectors can be calculated through various distance measures such as number of positions the vector has similar values excluding 0 [2]. Our implementation results in the output string that is the representation of the pattern the genes have undergone during transition from one state to another. Any gene can be queried against all the other genes by matching stored output strings in the database, and a 'score' is generated representing the similarity index between any given set of genes.

Affymetrix™ provides a calculation of absolute signal values for each gene for a given set of experiments, which can be viewed as points in n-dimensional space (where n is the number of experiments) [12]. Similarity between point representations of genes can be calculated using various metrics like Pearson correlation or Euclidean distance using various clustering algorithms [13]. Most databases (e.g. Comprehensive Systems-Biology Database (CSB.DB) [14] and the Arabidopsis Microarray Database and Analysis Toolbox (GENEVESTIGATOR) [15] help to seek shared biological roles based on correlation. These databases utilize existing methods like Pearson correlation and Spearman rank correlation to measure coregulation. These clustering methods provide reliable information for performing *internal comparison *of experimental conditions. But the usage of these for *cross comparisons *of various groups/clusters obtained through clustering various experimental conditions tends to obscure information important for identification of coregulated genes [8]. This implies that the clustering methods, which have shortcomings in identifying all the relationships existing in microarray expressions and different algorithms, will identify unique relationships thereby limiting them to the constraints of conditional coregulation [8,5]. Fuzzy k-means clustering implementation recognizes the concept of conditional coregulation and assigns 'membership' to each gene belonging to various clusters/groups [3].

We tried to incorporate the conditional coregulation for calculating relationships among genes by mining transcriptional data consisting of experiments in various conditions from AtGenExpress. For each condition in a temporal microarray experiment, the state of the gene at a particular time point is defined by alphabet according to the algorithm. The individual output string comprising of concatenated alphabets is stored on a per gene, per condition basis, meaning the length of the string equal to the number of time points for a particular experimental condition. Therefore, the number of alphabets and the complexity of a generated string for the given temporal experiment increases with an increase in number of timepoints. This implies that the algorithm performs better and produces more reliable results as the number of timepoints increase in the experiment. We tried to further reduce random matching of similar alphabets (at the same timepoint) by introducing an option to award extra increments if the preceding alphabet is matched.

## Methods

### Microarray datasets

The dataset consists of a total of 18 groups of experiments which were already preprocessed in MAS5.0  taken from aboveground samples of the abiotic stress series of microarray experiments conducted by AtGenExpress .

### Test data

The data consists of two sets of enzymes, i.e. nucleotide sugar interconversion enzymes [16] and glycosyltransferases , hypothesized to be involved in cell wall biosynthesis which consists of 493 genes for analyzing results.

**Figure 1:**
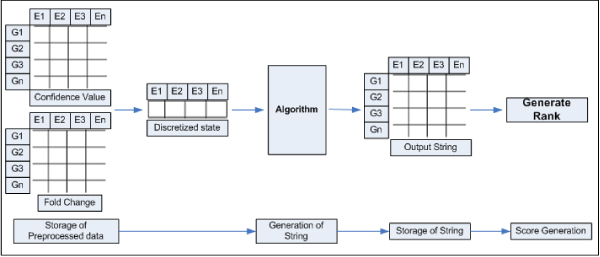
**Overview of the database construction**. E_1_, E_2_, E_3_, E_n _is the representation of the time points in temporal experiments. G_1_, G_2_, G_3_, G_n _is the representation of the gene. Confidence values and fold change for relative gene expression generated from Cyber-T.

The overview of the entire analysis and database construction is shown in Figure 1.

## Setting up database

### Storage of preprocessed data

We utilized Cyber-T  to measure the confidence value associated with fold change for each gene. The Cyber-T analysis (Control versus Experiment) uses Bayesian probabilistic framework to calculate a background variance for each of the genes under analysis. By combining the empirical variance with the local background variance associated with neighboring genes, it calculates the confidence associated with the differential expression [17,11]. This is supposed to compensate for the limited number of replicates by giving proper estimates of variances (which might be biologically relevant).

### Generation of string

The input matrices f_1_f_2_f_3_f_n _for fold change and p_1_p_2_p_3_p_n _for confidence value are generated from the analysis of Cyber-T, for each Control + Experiment. The fold change is the ratio of expression value between experiment and control, and negative fold change indicates lower expression in the experiment and vice versa. Discretization is utilized to reduce the variables in sample space resembling *"lossy compression method for the data*" [8]. Relative gene expression of every gene in each column (control plus experiment) is discretized into three distinct levels of differential expression – overexpressed, underexpressed and not expressed. These levels of differential expression are validated by the following rules:

i. An upregulated gene with a fold change (Expression_Experiment_/Expression_Control_) greater than +1 and *p *value less than the threshold is deemed as overexpressed and represented as positive state.

ii. A downregulated gene with a fold change (Expression_Control_/Expression_Experiment_) less than -1 and *p *value less than the threshold is categorized as underexpressed and represented as negative state.

iii. A gene with p value above the threshold is categorized as not expressed and represented as neutral state (0).

The algorithm utilizes an assumption that genes in a time series do not occupy discrete, independent expression devoid of any relationship with their previous experiments in a temporal series. In fact, the present state of each gene is dependent on its immediate preceding *state or vector *for magnitude and therefore generates an output string utilizing the graph theory [18] to depict a pattern. The algorithm uses these three levels of discretization to set the present state of each gene based on present and previous states.

The implementation of the algorithm is discussed as pseudo code below. The initial vector (state) of the gene is set by the above rules of discretization, which states the direction is positive or negative based on the level of expression and magnitude, is equal to the number of timepoint experiments as in line 6. Lines 10 to 21 of the algorithm describe the transition of genes in a timepoint experiment with resulting variable 'out'. The out variable in the algorithm is based on both the direction and magnitude of its previous state and present state as in lines 12, 14, 17 and 19. Each 'out' variable can be considered as an alphabet representing the state of the gene and stored in the matrix on a per gene, per experiment basis.

### Implementation of the algorithm

**a) i **is the number of the gene in the row

**b) j **is the number of the column (experiment)

**c) |len| **is the initial output assigned to the output string equal to number of time points for time series experiments

**d) state **is level of differential expression, i.e. overexpression, underexpression or no expression for a gene at a timepoint

**e) out **is the output alphabet for the transition of each gene i in j^th ^column

**f) PV(i, j) **is the matrix of confidence value(Bayes P) of i gene in j^th ^column

**g) FC(i, j) **is the matrix of Fold Change of i gene in j^th ^column

**h) DM(i, j) **is the matrix storing the output alphabet (out) as string

**i) threshold **is the user defined Bayes P value cutoff which has been taken as 5% arbitrarily

1. iterate for each gene: 0 to i {

2. iterate for each time series experiment: 0 to j {

3. if (PV(i, j) <= threshold) {

4. if (previous state !exists) { # true for first column

5. if (present state = (overexpressed | underexpressed)) {

6. out = ± len; DM(i, j) = out; } #+ overexpression, - underexpression

7. else if (present state = (not expressed)) {

8. out = 0; DM(i, j) = out; } }

9. else if (previous state exists) {

10. if (present state = previous state) {

11. if (FC(i, j) > FC(i, j-1) {

12. increment out; DM(i, j) = ± out; } #direction (±) based on present state

13. else {

14. decrement out; DM(i, j) = ± out; } }

15. else if (present state != previous state) {

16. if (FC(i, j) > FC(i, j-1) {

17. increment out; DM(i, j) = +out; }

18. else {

19. decrement out; DM(i, j) = -out; } }

20. } # 9 ends

21. } # 3 ends

22. else {

23. out = 0; DM(i, j) = out; }

24. } } # 1 ends

Note: # is the comment

The total number of alphabets generated for a time series n is 3+4^(n-1)^, and the number of strings (complexity) resultant from these alphabets for a time series n greater than 1 is 3^(n-1)^+2^(2n-1)^. For each individual experiment, a seperate string is generated with length equal to the timepoint of each experiment. These input values produce sequences of transition of a gene resulting in an output string o_1_o_2_o_3_...o_n _representing the expressional changes a gene has undergone that are stored in the database.

### Storage of generated string

The strings generated from the algorithm, for the time series experiment under different experimental conditions are stored in a RDBMS database. Each individual string is generated for a particular gene in a particular time series experiment and is stored separately.

### Generating a score through querying

The score is generated by matching strings of query gene with all the other genes in the database. For each experiment, the string of query gene is compared with strings of other genes, per experiment. The comparison is performed for all the experiments and aggregate score is computed. The string matching comprises of two determinants as follows:

#### Match

An alphabet of query string matching with alphabet of other genes at same temporal point of a time series experiment is awarded a unit.

#### Weight

Discretization leads to generalization of entire data. To overcome this aspect, any random similarity of the output alphabet is checked by further awarding an extra unit to any matching output alphabet having a preceding match. Weight provides an additional thrust to seek for genes undergoing similar patterns by separating them from genes with random similarity.

$$\text{Score}=\sum_{1}^{E}\sum_{1}^{T}Gn$$

T: is the number of time series experiments

E: is the number of experiments conducted in various conditions

Gn: is the value calculated in relation to a gene against a query gene using match and weight factors.

The web interface ASIDB at present defaults match and weight as a unit. The standalone java interface with a local database has a more dynamic interface allowing the user to enter these variables.

## Results and discussion

Genes represented in the ATH1-1250 chip can be queried based on Affymetrix™ Probeid or AGICode [1]. We utilised carbohydrate biosynthesis genes as a test case and at present, the database (hosted at ) refers to 493 genes annotated as glycosyltransferases and nucleotide sugar interconversion enzymes for *Arabidopsis thaliana*. The 'scores' generated by querying the database are retrieved in descending order of 'score' which we refer as 'rank' and used synonymously with 'score' for discussion. We have classified the results into two sections:

### A. Comparison of results with correlation coefficients derived from CSB.DB

#### Single gene query

We queried QUA1 (At3g25140) from our database and listed the top 15 genes (Table 1). We merged our results with CSB.DB by utilizing parametric Pearson's linear product moment correlation coefficient and the output using positive co-responding genes with probability < 0.05 by performing single gene query for QUA1 for atge0200 dataset. We found that many genes which generate high scores in ASIDB also have high confidence and high pearson in CSB.DB. Also, our results do not exclude genes unlike CSB.DB for the same dataset giving a more comprehensive picture for better analysis.

**Table 1:** Comparison of ASIDB score with CSB.DB for single gene. Descending order of score from ASIDB compared with the pearson correlation and the p value from CSB.DB for QUA1 for atge0200

Agicode	pearson coeff.	p value	Rank	Score	Name	Cazy Group
At5g60920	.6424	3.15e-08	1	126	COB	-
At2g20370	.8077	6.44E-15	2	123	MUR3	GT47
At2g47650	.3248	.0113	3	116	AUD2 UXS4	-
At3g29360	.7636	0.00000000000129	4	116	UGD2	-
At5g15490	.4858	0.0000833	5	112	UGD3	-
At1g19360	**	-	6	112	-	GT74
At4g22580	.3035	0.0184	7	111	-	GT47
At1g80290	**	-	8	110	-	GT64
At5g39320	.3551	0.00537	9	110	UGD1	-
At1g16900	.3636	0.0043	10	109	-	GT22
At3g23820	.7862	9.88E-14	11	109	GAE6	-
At2g22900	.6505	0.0000000185	12	109	-	GT34
At1g53500	.6859	0.00000000147	13	109	RHM2	-
At1g08660	**	-	14	105	-	GT29
At1g06000	**	-	15	104	-	GT1

** Genes not present in CSB.DB (atge0200 matrix)

### Subgroup (five UGE isoforms) comparison

Performing co-response analysis for UGE isoforms by comparing fluctuation of transcript abundance between CSB.DB (Spearman correlation) and ASIDB revealed UGE1 and -3 behaved differently than UGE2, -4, -5 [19].

### Random comparison of genes from CSB.DB

**Figure 2:**
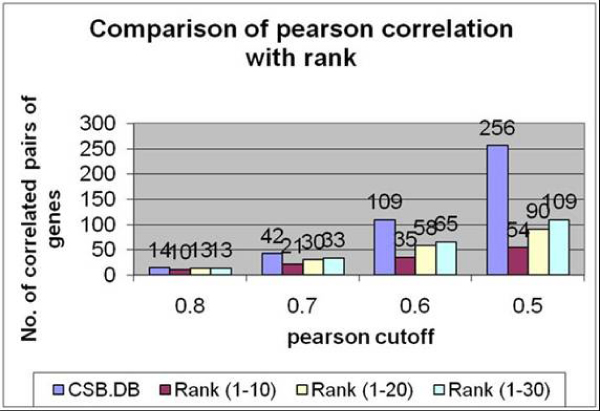
**Comparison of pearson correlation with ASIDB score**. Graph depicts the number of correlated pairs at different pearson cutoff and the number of those same correlated pairs identified by ASIDB at different rank cutoff.

We randomly selected around 10% of the genes (~52 genes) from our database and compared them with Pearson correlation generated from CSB.DB. The correlation coefficient measures the relationship between two variables ranging between +1 and -1 where +0.7 to +1.0 is considered strong positive association, +0.3 to +0.7 as weak positive association and +0.3 to 0 as no association [20]. Comparing 1326 correlated pairs generated from CSB.DB and ASIDB, we found that at high pearson correlation, i.e. .8, out of 14 correlated pairs generated by CSB.DB, 10 were identified by ASIDB at rank cutoff of 10 genes (top10 genes) and 13 correlated pairs at rank cutoff of 20 (Figure 2). Similarly for pearson correlation cutoff at .7, we were able to identify half (21) of the correlated pairs at rank cutoff of 10 and 30 at rank cutoff of 20. We observe the trend that ASIDB identifies most of the correlated pairs at high pearson value and identification reduces with declining pearson for the same genes. ASIDB also generates a higher number of relationships that are not identified by pearson correlation and might hold biological importance. The comparison file can be downloaded from .

### B. Biological validity of the results

We utilized genes hypothesized to be involved in cell wall biosynthesis (glcosyltranferases and nucleotide interconversion enzyme) as test data. Out of these, around 420 genes that are present in carbohydrate active enzymes  are glycosyltransferases while the remaining genes are linked with nucleotide sugar interconversion enzymes. The glycosyltransferases are specific for both the donor sugar nucleotide and the acceptor molecule, which might be another sugar or aglycones. One glycosyltransferase usually catalzses the formation of only one glycosidic linkage. As a result, though many glycosyltransferases catalyze chemically similar reactions, they display remarkable diversity in their donor, acceptor and product specificity and thereby generate a potentially infinite number of glycoconjugates, oligosaccharides and polysaccharides [21]. The high specificity of glycosyltransferases results in difficulty in assessing the biochemical function of the enzymes encoded by these genes [22]. Furthermore, the application of clustering or any other technique has not yielded a precise donor, acceptor or product specificity of glycosyltransferases [23].

The activated sugars, known as nucleotide sugars, form the substrates for nucleotide sugar interconversion enzymes dedicated to the generation of new sugar species [24]. The biochemical and molecular aspects of sugar nucleotide interconversion enzymes are fairly well understood [25]. Also, nucleotide sugars are the substrates for glycosyltransferases that catalyze the polymerization of monosaccharides into glycosides, oligosaccharides, glycolipids, glycogen, starch, cellulose and a large variety of extracellular complex carbohydrates [24,26]. An improved description of the link between nucleotide sugar interconversion genes and glycosyltransferases might help in understanding the control of cell wall biosynthesis [16].

### Relationship of UGE with glycosyltransferase

On querying five UGE isoforms (Table 2), we subgrouped them into two groups based on their relationship with each other and glcosyltransferases MUR3 (At2g20370), GOLS2 (At1g56600) and ATGT18 (At5g62220).

**Table 2:** Comparison of ASIDB ranking with pearson correlation for UGE homologs. The ranking of genes depicting relationship between UGE homologs and glycosyltransferases with pearson correlation is in parenthesis.

RANK	MUR3	AtGT18	UGE4	UGE2	UGE5	UGE1	UGE3
UGE1	477 (.4408)	460 (.2894)	478 (.2306)	473 (.3102)	374 (NA)	-	10 (.6303)
UGE2	14 (.5818)	42 (.2842)	97 (.1159)	-	9 (NA)	473 (.3102)	340 (.6475)
UGE3	474 (-.424)	461 (.2785)	404 (.1084)	340 (.6475)	402 (NA)	4 (.6303)	-
UGE4	1 (.0748)	291 (.3508)	-	131 (.1159)	43 (NA)	483 (.2306)	431 (.1084)
UGE5	22 (NA)	57 (NA)	52 (NA)	13 (NA)	-	365 (NA)	409 (NA)
MUR3	-	63 (.3995)	13 (.0748)	46 (.5818)	66 (NA)	484 (.4408)	482 (-.424)
QUA	2 (.8077)	159 (.1981)	35 (.1153)	118 (.5349)	226 (NA)	447 (.3241)	486 (.3395)
GOLS2	34 (NA)	46 (NA)	51 (NA)	1 (NA)	8 (NA)	465 (NA)	324 (NA)

*NA represents genes omitted in the CSB.DB (atge0200 matrix)

i) UGE1, -3

UGE1 and UGE3 co-regulated with trehalose 6-phosphate synthases [27] indicating their catabolic role [19,28].

ii) UGE2, -4, -5

We observed that UGE2,-4 and -5 co-regulated with known galactosyltransferases like MUR3, GolS2, ATGT18 indicating biosynthesis role [19,28].

### Relationship among cellulose synthases

The Arabidopsis genome encodes 10 isoforms of the cellulose synthase catalytic subunit – CESA [29] and we have broken down these groups of genes into two sets based on the rank derived from Table 3.

**Table 3:** Comparison of ASIDB ranking with pearson correlation for CESA homologs. The ranking of genes depicting relationship between CESA subgroup derived from the score with the pearson correlation in parenthesis.

RANK	CESA1	CESA2	CESA3	CESA5	CESA6	CESA4	CESA7	CESA8
CESA1	-	53 (.4319)	1 (.8554)	25 (.4822)	2 (.7231)	155 (.3368)	152 (.2495)	439 (.3224)
CESA3	2 (.8554)	9 (.5283)	-	7 (.5252)	5 (.7179)	312 (.1678)	219 (.1093)	350 (.1853)
CESA6	3 (.7231)	14 (.5684)	17 (.7179)	1 (.6678)	-	240 (.1605)	125 (-.0458)	365 (-.033)
CESA4	165 (.3368)	453 (-.2676)	368 (.1678)	287 (.022)	231 (.1605)	-	1 (.6776)	8 (.5777)
CESA7	118 (.2495)	376 (-.4548)	242 (.1093)	282 (-.0267)	73	1 (.6776)	-	2 (.7249)
CESA8	448 (.3224)	193 (-.186)	397 (.1853)	382 (.046)	323 (-.033)	4 (.5777)	1 (.7249)	-

*NA is genes omitted in the CSB.DB (atge0200 matrix)

i) AtCesa1, AtCesa3, AtCesa6, AtCesa5, AtCesa2

Cesa1,-3,-6 are responsible for cellulose production during primary cell wall development in various tissues [30]. On querying ASIDB, we found that not only CESA2 which have been earlier found to be highly co-regulated with CESA1,-3,-6 came at higher rank [30], but CESA5 also shows co-regulation with this set of genes indicating its role in deposition of cellulose in the primary cell wall.

ii) AtCesa4, AtCesa 7, AtCesa 8

The irx1, irx3 and irx5 mutants are the members of CesA gene family and AtCesA4 (IRX5), AtCesA7 (IRX3) and AtCesA8 (IRX1) take part in the synthesis of the complex that is required to synthesize cellulose in the secondary cell wall [30,31]. Other genes, which have shown significant relationship with these genes and have also been mentioned in previous work, are At5g54690, At2g37090 [30].

## Conclusion

We tried to incorporate the conditional co-regulation for calculating the relationship among genes by addressing the limitation of clustering methods in which genes that are expressed in most of the measurements are highlighted while genes that are co-expressed in the subset of conditions are omitted. Our implementation leads to the inclusion of every gene regardless of its expression values thereby highlighting an important relationship absent in other contemporary databases. We found that our approach not only recognizes most of the randomly selected correlated pairs from pearson correlation in CSB.DB, but also generates new relationships. This has resulted in highlighting subtle relationships for example UGE isoforms [19,28].

**Figure 3:**
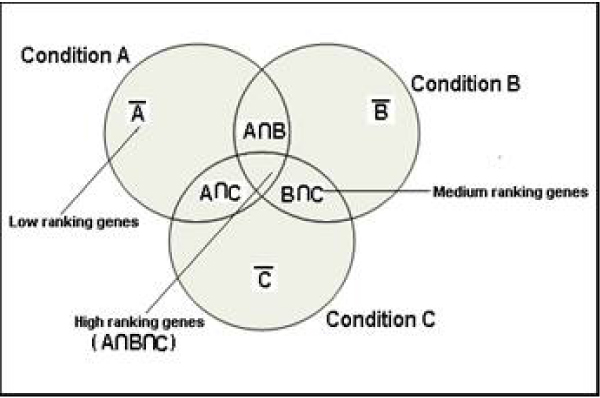
**Generation of scores from different experimental conditions**. The generation of a score resulting from the overlapping of similar expressional patterns under varying conditions with the query gene.

**Figure 4:**
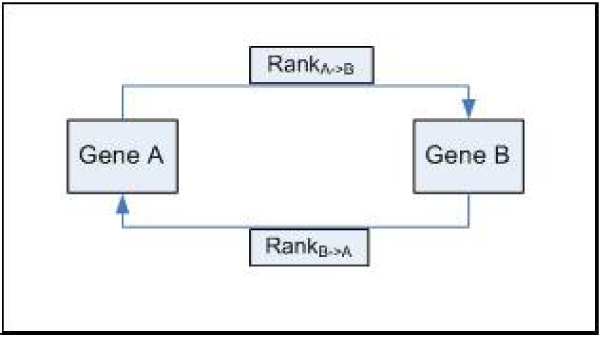
**Depiction of network relationship between genes**. The bi-directional relationship generated between gene A and gene B connected by edges. The edges represent score/rank by performing querying for each gene.

Taking carbohydrate metabolism as a test case, we observed that those genes known to be involved in similar functions and pathways generate a high 'score' with the queried gene. The 'score' is therefore computed for all the genes present in the database and reflects the magnitude of co-regulation existing with the queried gene. The higher the intersection of expressional patterns under varying conditions, the higher the score generated for the gene calculated by the algorithm (Figure 3) and ranked in descending order of 'score'. Interpretation of the results is done by considering genes as nodes linked with each other through the edges [32]. Edges represent interactions between the connected genes, with higher rank/score depicting higher functional similarity (Figure 4). We can utilize the interaction of edges and nodes for the construction of the networks.

After implementing the carbohydrate biosynthesis cycle, we intend to incorporate other cycles like amino acid, nucleotide, and lipid. We have recently added ARACYC  annotation to our database.

## Competing interests

The authors declare that they have no competing interests.

## Authors' contributions

AR conceived the datamining project, wrote the algorithm, created the database, initiated ASIDB and wrote the manuscript. YD coordinated the project and revised the manuscript. All authors read and agree to publish the manuscript.
